# The Effect of the EGFR - Targeting Compound 3-[(4-Phenylpyrimidin-2-yl) Amino] Benzene-1-Sulfonamide (13f) against Cholangiocarcinoma Cell Lines

**DOI:** 10.31557/APJCP.2021.22.2.381

**Published:** 2021-02

**Authors:** Papavee Samatiwat, Lueacha Tabtimmai, Prapasri Suphakun, Nattanan Jiwacharoenchai, Borvorrnvat Toviwek, Veerapol Kukongviriyapan, M. Paul Gleeson, Kiattawee Choowongkomon

**Affiliations:** 1 *Department of Pharmacology, Faculty of Medicine, Srinakharinwirot University, Bangkok, 10110, Thailand. *; 2 *Department of Biochemistry, Kasetsart University, Bangkok, 10900, Thailand. *; 3 *Genetic Engineering Interdisciplinary Program, Graduate School, Kasetsart University, 10900, Thailand. *; 4 *Department of Chemistry, Kasetsart University, Bangkok, 10900, Thailand. *; 5 *Department of Pharmacology, Faculty of Medicine, Khon Kaen University, Khon Kaen, 40002, Thailand. *; 6 *Cholangiocarcinoma Research Institute, Khon Kaen University, Khon Kaen, 40002, Thailand.*; 7 *Department of Biomedical Engineering, Faculty of Engineering, King Mongkut’s Institute of Technology Ladkrabang, Bangkok. Thailand. *; 8 *Center for Advanced Studies in Nanotechnology for Chemical, Food and Agricultural Industries, KU Institute for Advanced Studies, Kasetsart University, Bangkok 10900, Thailand. *

**Keywords:** EGFR, cholangiocarcinoma, chemotherapy, antiproliferation

## Abstract

**Objective:**

Cholangiocarcinoma (CCA) is a noxious malignancy of epithelium of the bile duct with a low response rate to chemotherapy. The epidermal growth factor receptor (EGFR) signaling pathway is implicated in the development of cancerous cells, especially CCA. In this study, we report detailed biological profiling of 13f identified from our earlier hit expansion studies. The aim of this work was to expand our understanding of 13f via more detailed investigations of its mechanism of action against KKU-100, KKU-452 and KKU-M156 CCA cells, as well as in comparison to the EGFR inhibitor Gefitinib and non-specific chemotherapeutic agents such as Cisplatin.

**Methods:**

Inhibiting EGFR-Kinase, cytotoxicity, clonogenic assay, wound healing and apoptosis were performed. Levels of total expression of EGFR and EGFR phosphorylation proteins were detected.

**Results:**

13f was confirmed as an inhibitor of EGFR with an IC_50_ value against the tyrosine kinase of EGFR of 22 nM and IC_50_ values for 48 h incubation period were 1.3 ± 1.9, 1.5 ± 0.4 and 1.7 ± 1.1 µM of KKU-100, KKU-452 and KKU-M156, respectively through dose- and time-dependent induction of early apoptosis of CCA cells. The compound also suppressed the clonogenic ability of KKU-100 and KKU-M156 cells stronger than Gefitinib, while potently inhibiting EGF-stimulated CCA cell migratory activity in KKU-452 cells. It was observed that under normal conditions EGFR was activated in CCA cells. EGF-stimulated basal expression of EGFR in KKU-452 cells was suppressed following 13f treatment, which was significantly greater than that of the marketed EGFR inhibitor Gefitinib.

**Conclusion:**

In summary, our study showed that 13f has potent anti-cancer activities including antiproliferation, clonogenic ability and migration through the modulation of EGFR signaling pathway in CCA for the first time. The compound represents an interesting starting point as a potential chemotherapeutic agent in ongoing efforts to improve response rate in CCA patients.

## Introduction

Cholangiocarcinoma (CCA) is the second most common of primary hepatic cancer that rinsing from epithelial of biliary duct (Everhart and Ruhl, 2009). The highest incidence rates of CCA were found Southeast Asia, particularly in the northeastern Thailand, and the lowest in Australia (Bridgewater et al., 2014; Blechacz, 2017). In recent decades, CCA incidence has significantly risen in Europe and North America (Khan et al., 2012b; Witjes et al., 2012; Tyson et al., 2014; Blechacz, 2017). A major risk factor associated with CCA is parasitic liver flukes, including *Opisthorchis viverrini* and *Clonorchis sinensis*, which parasite infestations result in chronic inflammation and irritation (Sriamporn et al., 2004; Nagino et al., 2013). Patients are commonly diagnosed at late stage with high recurrent cancers after surgery, resistance to cancer chemotherapy and poor prognosis (Khan et al., 2012a). In fact, 5-year survival rates following effective curative surgery are reported to be only 25%-30% (Nagino et al., 2013). Chemotherapy is one of the treatment choices for unresectable CCA patients. However, an important obstacle for treating CCA is the resistance to chemotherapy. Clinical trials have shown a median overall survival of only 11.7 months after treatment with the standard chemotherapy cisplatin and gemcitabine (Valle et al., 2010; Bridgewater et al., 2016). Accordingly, several studies have revealed different mechanisms of chemo-resistance such as cytoprotective pathway stimulation (Samatiwat et al., 2015; Samatiwat et al., 2016), AMPK-mTOR cellular metabolism pathway alteration (Wandee et al., 2018), and epidermal growth factor receptor (EGFR) signal activation (Yoon et al., 2004; Abachi et al., 2017). 

EGFR is receptor tyrosine kinase (RTK) which is activated by its ligand growth factors such as epidermal growth factor, heparin binding epidermal growth factor-like growth factor, transforming growth factor-alpha, insulin-like growth factor and hepatocyte growth factor (Bogdan and Klambt, 2001; LeRoith and Roberts, 2003; Jiang et al., 2005). EGFR is linked to many downstream pathways which contribute to its role in cell differentiation, migration, proliferation and survival (Zaczek et al., 2005; Verma et al., 2012). Dysregulation and overexpression of the EGFR-signaling pathway in several cancers ameliorate carcinogenesis (Ritter and Arteaga, 2003), angiogenesis (Minder et al., 2015), cancer progression (Franklin et al., 2002; Yoon et al., 2004) and cellular survival (Yang et al., 2014). EGFR inhibitors consist of small molecule tyrosine kinase inhibitors of the catalytic domain including Gefitinib, Erlotinib and Lapatinib along with monoclonal antibodies that inhibit EGFR downstream signaling via bindings to the extracellular domain including Cetuximab and Panitumumab (Goss et al., 2009; Nie et al., 2009; Jiang et al., 2013; Zhao et al., 2014; Qi et al., 2015; Lee et al., 2017). EGFR targeted therapies have been approved as monotherapies and combination therapies in diverse cancers such as non–small-cell lung cancer (Shepherd et al., 2005), pancreatic cancer (Moore et al., 2007), colorectal cancer (Kennecke et al., 2013), breast cancer (Nelson and Dolder, 2006) and head and neck cancer (Baba et al., 2012). 

In CCA, an overexpression of EGFR has been reported to be one of the main prognostic factors (Yoshikawa et al., 2008; Yang et al., 2014). In vitro and in vivo studies found that the constitutive activation of EGFR signaling pathway is potentiated in EGFR targeted therapies (Yoon et al., 2004; Yoshikawa et al., 2009; Xu et al., 2010). However, the efficacy of EGFR targeted therapies in clinical trials has yet to be conclusively proven (Philip et al., 2006; Paule et al., 2007). EGFR targeted therapies were used in combination with Gemcitabine-based therapy giving rise to a median overall survival of 7–12.9 months (Paule et al., 2007; Gruenberger et al., 2010; Jensen et al., 2012; Lee et al., 2012; Sohal et al., 2013; Malka et al., 2014). Furthermore, a case report was recently published in which the use of EGFR inhibitors was shown to be more effective in CCA patients than standard therapies based on the longer than median survival times observed (Poddubskaya et al., 2018). Limitation associated with EGFR targeted therapies nevertheless exist, including chemoresistance. In the clinical trials also reveal a potential use of panitumumab anti-EGFR combined to gemcitabine and oxaliplatin (GEMOX) chemotherapy but it did not show to improve the overall survival of CCA patient (Peraldo-Neia et al., 2018). Other EGFR-targeted inhibitors were also used as alternative monotherapy and combination with cytotoxic agents therapy and developed for CCA also not improve survival in CCA patients in large clinical studies (Philip et al., 2006; Ramanathan et al., 2009; Gruenberger et al., 2010; Zhu et al., 2010). This necessitates the continuing identification of new inhibitors that can overcome or at the very least reduce CCA resistance. 

This present study was undertaken following earlier hit to lead studies of our group which saw the preparation and antiproliferation assessment of 20 4-aryl-N-phenylpyrimidin-2-amines (Toviwek et al., 2017). The anti-proliferation activity of the set was assessed in the EGFR-overexpressing A549 cell-line leading to the identification of 13f as a sub-micromolar inhibitor with good physico-chemical properties. However, the most likely therapeutic target or the stage at which cell death was occurs was not investigated. The goal of this work was to shed light on the mode of action of this inhibitor and to assess the extent of its activity across a range of different cell lines not previously investigated. Furthermore, we were interested in understanding whether EGFR kinase played a role in the inhibition process and understanding the stage at which cell death was being promoted. Finally, we compared and contrasted the mode of inhibition of 13f with a potent EGFR inhibitor Gefitinib and a non-specific chemotherapeutic agent cisplatin.

## Materials and Methods

*Reagents*

The cell culture medium Ham-12, fetal bovine serum (FBS), dimethylsulfoxide (DMSO), and streptomycin-penicillin reagents were purchased from Gibco BRL Life Technologies (Grand Island, NY, USA). MTT [3-(4,5-dimethylthiazol-2-yl)-2,5-diphenyltetrazolium bromide], EGF and Gefitinib were purchased from Sigma-Aldrich Inc. (Saint Louis, MO, USA). Cisplatin was obtained from Boryung Pharm (Seoul, South Korea). ADP-Glo™ from Promega (Madison, USA). 13f was prepared as described elsewhere by us (Toviwek et al., 2017).

*Inhibiting EGFR-Kinase assay*

The assay reaction was done by using the ADP-Glo™ Kinase Assay kit. The tyrosine kinase of EGFR (TK-EGFR), a recombinant protein was obtained from E.coli expression system. The 6.5 ng of TK-EGFR protein was incubated with serially diluted of 13f inhibitor in 384-well white flat bottom polystyrene plates. After that, the reaction was added substrate with 5 µM of ATP and 2 µg/ml of PolyTry:glu and incubated for 1 h at room temperature. The reaction was terminated using 5 µl of ADPGlo, then incubated for 40 mins at room temperature. And then, the reaction was added 10 µl, and incubated 30 mins at room temperature. Finally, the reaction was measured ATP. The light generated was measured using a luminescent by Synergy HTX Multi-Mode Reader (BioTek, UK). Graphpad Prism software (version 6.0) was used for IC_50_ analysis.

*Cell cultures*

The human CCA cell lines were used in this study including, KKU-K452, KKU-M56 and KKU-100. KKU-452 cells were kindly prepared from Prof. Dr. Veerapol Kukongviriyapan, Department of Pharmacology, Faculty of Medicine, the Cholangiocarcinoma Research Institute, Khon Kaen University. KKU-M56 and KKU-100 cells were kindly provided from Prof. Banchob Sripa, Department of Pathology, Faculty of Medicine, Khon Kaen University. The CCA cell lines were established and derived from tumor tissue of Thai CCA patients (Sripa et al., 2005; Yonglitthipagon et al., 2010; Saensa-Ard et al., 2017). The culture medium is Ham’s F12 with 12.5 mM N-2-hydroxyethylpiperazine-N’-2-ethanesulfonic acid (HEPES), pH 7.3, 100 U/ml penicillin, 100 µg/ml streptomycin and 10% fetal bovine serum (FBS). The cells were maintained at 37°C with 5% CO2 and sub-cultured every 2-3 days using 0.25% trypsin-EDTA as the previously described (Samatiwat et al., 2016).

*Cytotoxicity*

KKU-K452, KKU-M56 and KKU-100 cells were used and seeded into 96-well plates at a density of 7.5 x 10^3^ cells/well an overnight incubation. The compounds for testing were dissolved in DMSO vehicle and diluted with complete medium at various concentrations. The final DMSO vehicle was 0.1 % in each experiment. After 24 and 48 h, the cytotoxicity was performed using the MTT assay as the previously described (Mosmann, 1983). Absorbance of the formazan product was measured at 540 nm using SpectraMaxM2 microplate readers. The percentage of cell cytotoxicity was calculated as 100-(absorbance of experiment / absorbance of control x 100). The IC_50_ value was determined by a nonlinear curve-fitting of SigmaPlot version 10 program. 

*Clonogenic assay*

The clonogenic assay was conducted according to the previously our method described (Samatiwat et al., 2016). KKU-100 and KKU-M156 cells were used at seeding density of 600 cells/well into a six-well culture plate. After 48 h, the complete medium was changed with 0.1, 1 µM of Gefitinib or 0.1, 1 µM of 13f for 24 h incubation time. Then, KKU-100 and KKU-M156 cells were continued in the complete medium another 8 days and replaced with fresh medium every 2 days. Crystal violet was provided for cells staining. At least 50 cells as the one colony were counted under the microscope examination. 

*Wound healing assay*

A wound healing method was executed according to previously reported (Wandee et al., 2018). KKU-452 cells were seeded at density of 2.5 x 10^5^ cells/well into a 24-well plate and allowed cell growth for 48 h in complete medium. Cells were scratched with a sterile pipette tip and washed a scratch wound with PBS solution to pull out debris and unattached cells. Cells were pretreated with 0.5 µM of Gefitinib or 0.5 µM of 13f or a vehicle treated control for 3 h and incubated with 5 ng/ml of EGF. The scratch wound was captured a series of pictures under the microscope from 0, 6 and 12 h. The measurement area of a scratch wound was analyzed using the Image-Pro Plus program (Media Cybemetrics, LP, USA). Cell migration rate was evaluated from an average width ration between the given time and the initial time.

*Annexin V apoptosis assay*

KKU-K452, KKU-M56 and KKU-100 cells were seeded at density of 2.5 x 10^5^ cells/well into a 6-well plate in complete medium. Cells were treated with Gefitinib or 13f at a concentration of 0.1, 1 and 10 µM for 24 and 48 h. Apoptosis was performed by MuseTM Annexin V & Dead Cell reagent (Merk Millipore, Germany). Cells were dissociated from each well to obtain single-cell suspensions then added 50 µl of MuseTM Annexin V and Dead Cell reagent. Each condition was mixed thoroughly by vortexing and was then stained in the dark for 20 min at room temperature. Cells were inspected by flow cytometry (Guava EasyCyte HT, Millipore, Bedford, MA, USA).

*EGFR-RTK Activation*

KKU-452 cells were seeded at density of 2.5 x 10^5^ cells/well into a 6-well plate and in complete medium. Cells were pretreated with 0.1 µM of Gefitinib or 0.1 µM of 13f or a vehicle treated control for 3 h and then incubated with 5 ng/ml of EGF for 24 h. The scratch wound was captured a series of pictures under the microscope from 0, 6 and 12 h. The expressions of total EGFR and EGFR phosphorylation protein levels were examined by MuseTM EGFR-RTK Activation Dual Detection kit (Merck Millipore, Germany) using an anti-phospho-EGFR (Tyr1173) – Alexa Fluor 555 for phosphor-form and an anti-EGFR – PECy5 according to the manufacture’s protocol. EGFR signaling activations were measured by the relative ratio of the EGFR phophorylation and the total EGFR expression using The MuseTM Cell Analyzer with the MuseTM software. 

*Statistical analyses*

Results were presented as the mean ± SEM for triplicate experiments. Statistical comparisons between control and treatment group were analyzed using Student’s t-test or ANOVA with Student-Newman-Keuls post hoc test, where appropriate. P < 0.05 was considered statistically significant.

## Results

*13f as a new inhibitor of EGFR*

**Figure 1:**
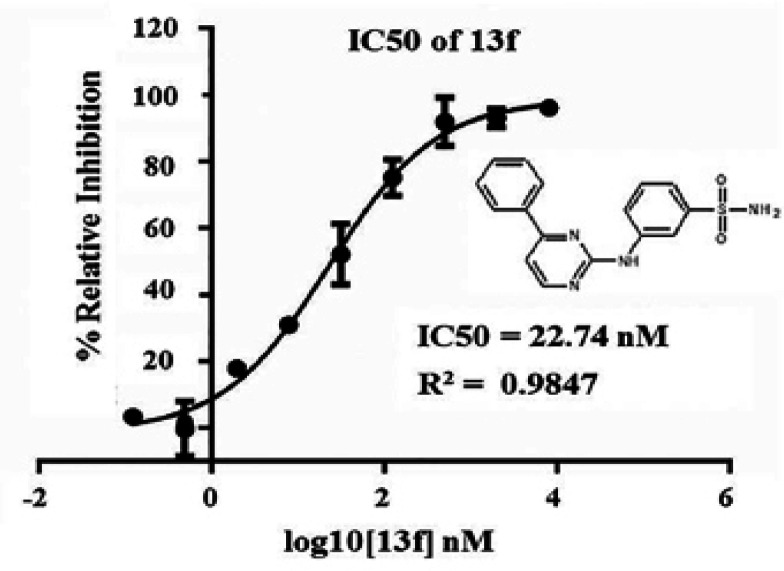
13f is an Inhibitor of the Tyrosine Kinase of EGFR. The tyrosine kinase of EGFR (TK-EGFR) assay was performed using the ADP-Glo™ Kinase Assay kit. Data represent the percent of EGFR relative inhibition of by 13f with the IC50 value analysis of three independent experiments. The structure of 13f was shown

13f is a new synthetic 3-[(4-phenylpyrimidin-2-yl) amino] benzene-1-sulfonamide and was prepared by us as described previous report (Toviwek et al., 2017). This study aims to confirm 13f as a new EGFR inhibitor using EGFR-kinase inhibiting assay. The result found that 13f can inhibit the tyrosine kinase of EGFR with the IC_50 _value is 22.74 nM (Figure1). 13f is a good potential for evaluate the anti-cancer activity of CCA which elevates the *EGFR* expression. 

*Cytotoxicity of tested agents*

The antiproliferative effects of Gefitinib (EGFR inhibitor), 13f (a new EGFR inhibitor) and Cisplatin (cytotoxic chemotherapy) were examined in KKU-100, KKU-452 and KKU-M156 cells using the MTT assay for 24 h and 48 h. The results demonstrated that these agents were toxic to all CCA cells in a time-dependent manner and indicated 100 % of percent of maximal cancer cell killing effect (Emax) for each agent as shown in Table 1. Gefitinib, 13f and Cisplatin displayed a strongly antiproliferative effect in KKU-100 cells after 48 h exposure with the IC_50_ values of 4.0 ± 1.4, 1.3 ± 1.9 and 18.1 ± 3.9 μM, respectively. 13f had the highest cytotoxicity against CCA cells. It should be noted that KKU-452 was highly sensitivity to 13f after 24 h exposure with the IC_50_ values of 4.2 ± 2.2. This data suggested that all CCA cells were resistant to Cisplatin as a standard chemotherapy in CCA patients. On the other hand, all CCA cells were good responsive to Gefitinib and 13f as an EGFR inhibitor. EGFR might play a crucial role in cytotoxicity in CCA. 

*Gefitinib and 13f induced apoptosis of CCA cells*

**Figure 2.**
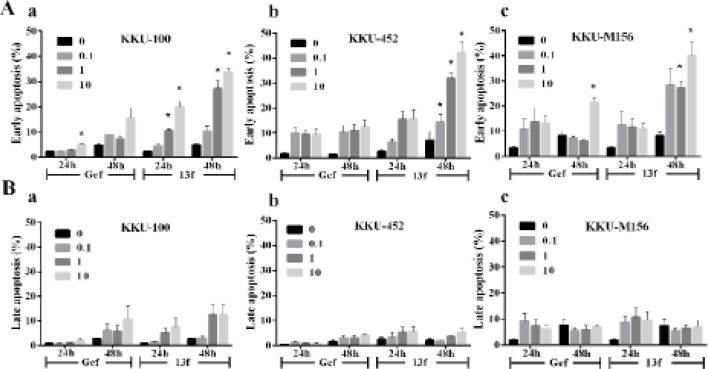
Effect of Gefitinib and 13f on the Mode of CCA Cells Death. KKU-100, KKU-452 and KKU-M156 cells were treated with 0.1, 1 and 10 μM Gefitinib (Gef) and 13f for 24 and 48 h. The cells were stained with Annexin V and analyzed by flow cytometry. A) The graph shows the percentage of early apoptosis of KKU-100 (a), KKU-452 (b), and KKU-M156 cells (c). B) The graph shows the percentage of late apoptosis of KKU-100 (a) KKU-452 (b) and KKU-M156 cells (c). Data indicate the mean ± SEM averaged from three independent experiments. Value of *P < 0.05 vs control (0) was indicated

To compare the effects of Gefitinib and 13f on the mode of cell death in KKU-100, KKU-452 and KKU-M156 cells were evaluated using flow cytometry analysis. The results indicated that CCA cells were remarkably altered in early apoptosis and slightly increased late apoptosis by Gefitinib and 13f after 24 and 48 h (Figure 2A-B). Gefitinib 10 µM triggered cytotoxicity with significantly induced early apoptotic cell death as 5.0 ± 1.0 % of KKU-100 for 24 h and 21.6 ± 3.1 % of KKU-M156 for 48h. KKU-100 cells were significantly induced early apoptotic cell death in a concentration-and time-dependent manner as 10.6 ± 0.9 and 20.0 ± 3.8% for 1 and 10 µM 13f after 24 h and significantly induced early apoptotic cell death increasing as 27.1 ± 5.5 and 33.8 ± 2.6 % for 1 and 10 µM 13f after 48 h. KKU-452 cells were significantly induced early apoptotic cell death in a concentration-dependent manner as 14.2 ± 5.5, 31.8 ± 4.2 and 42.4 ± 7.0 % for 0.1, 1 and 10 µM 13f after 48 h. KKU-M156 cells were significantly induced early apoptotic cell death in a concentration-dependent manner as 27.13 ± 4.8 and 40.1 ± 9.1 % for 1 and 10 µM 13f after 48 h. It can therefore be concluded that 13f exhibited stronger potency than Gefitinib in triggering cytotoxicity by induced early apoptosis in a dose- and time dependence. 

*The effects of Gefitinib and 13f on colony formation of CCA cells*

**Figure 3:**
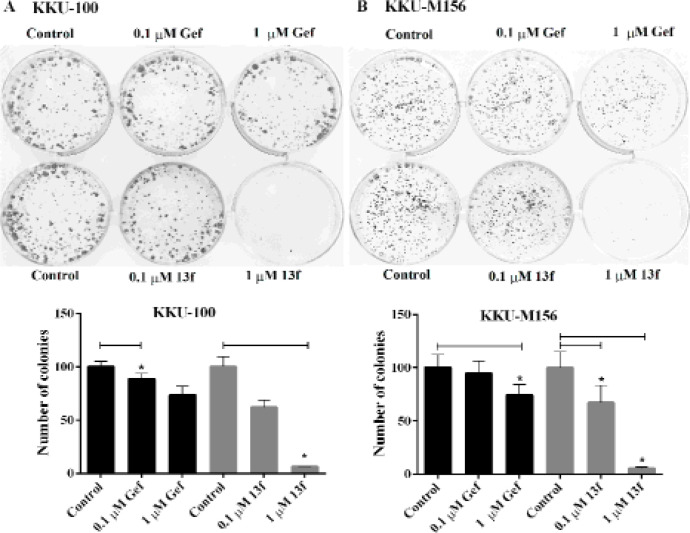
Effects of Gefitinib and 13f on Colony Forming Ability of CCA Cells. KKU-100 and KKU-M156 cells were treated with 0.1 and 1 μM Gefitinib (Gef.) or 0.1 and 1 μM 13f for 24 h in 6-well plates, then replaced with complete culture medium. After 8-days, cells were stained with crystal violet and colony formation was photographed. A) KKU-100 cells were analyzed for the colony formation. B) KKU-M156 cells were analyzed for the colony formation. Figures shown are one of three similar experiments. The graph indicates the percentage of a number of colonies formation relative to the controls and represents the mean ± SEM averaged from three independent experiments. Value of *P < 0.05 vs control was indicated

Next, the effects of Gefitinib and 13f on colony forming ability using clonogenic assay were examined. Gefitinib and 13f could suppress colony forming ability both of KKU-100 and KKU-M156 cells in a concentration dependent manner (0.1 and 1 μM) (Figure 3A-B). In KKU-100 cells, the significant inhibitory effect was shown at the concentration of 0.1 μM Gefitinib with a number of colonies as 88.6 ± 9.1 (11.4%), and 1 μM 13f with a number of colonies as 6.2 ± 0.8 (93.8%) when compared with control group. In KKU-M156 cells, the significant inhibitory effect was shown at the concentration of 1 μM Gefitinib with a number of colonies as 74.21 ± 17.5 (25.8%) together with 0.1 and 1 μM 13f with a number of colonies as 67.1 ± 28.0 (32.9%) and 22.44 ± 2.2 (77.6%), respectively when compared with control group. Gefitinib and 13f were better to suppress colony forming ability of KKU100 cells than KKU-M156 cells. The results suggested that replicative ability of CCA cells was inhibited by Gefitinib and 13f. Moreover, the efficacy of 13f was stronger than Gefitinib to suppress replicative ability of CCA cells.

*The effects of Gefitinib and 13f on CCA cell migration*

**Figure 4:**
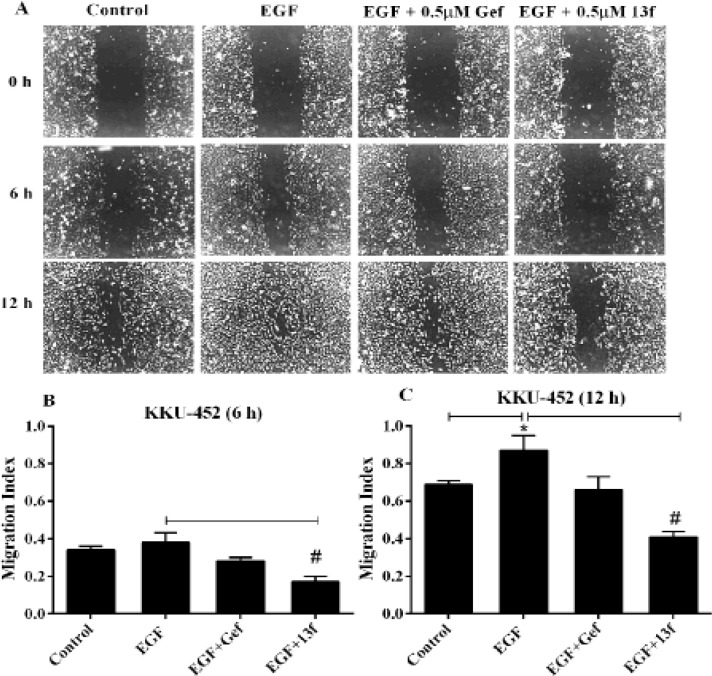
The Effects of Gefitinib and 13f on CCA Cells Migration. KKU-452 Cell Migration was Performed by Wound Healing Assay. Cells were scratched and pretreated with 0.5 μM Gefitinib (Gef), 0.5 μM 13f and a vehicle treated control for 3 h, then 5 ng/ ml EGF was added and incubation period to 12 h. A) A series of images of the scratched wound were taken from 0, 6 and 12 h under phase contrast microscopy (10 X magnifications). B) The bar represent means ± SEM of migration index at 6 h. Migration index indicating the level of cell migration was calculated by the ratio of net wound width at the given time and the initial time. C) The bar represent means ± SEM of migration index at 12 h. Each bar averaged from 9 separated areas of three independent experiments. Value of *P < 0.05 vs control or # P < 0.05 vs EGF were indicated

EGFR has an important role in enhanced migration of cancer cell. Thus, we evaluated the effects of Gefitinib and 13f on CCA cell migration underlying EGF stimulation using wound healing assay. KKU-452 cells were significantly closed wound by EGF stimulation at 12 h with migration index was 0.88 (Figure 3A and C). We found that 0.5 μM Gefitinib could retard KKU-452 cell migration underlying EGF stimulation but no statistical different (Figure 4A, B and C). 13f of 0.5 μM had a significant inhibitory effect on KKU-452 cell migration underlying EGF stimulation both of 6 and 12 h with migration index was 0.17 and 0.42, respectively (Figure 3A, B and C). These data indicated that 13f could be potently inhibited EGF stimulated CCA cell migratory activity.

*The effect of Gefitinib and 13f on EGFR expression of CCA cell*

**Figure 5:**
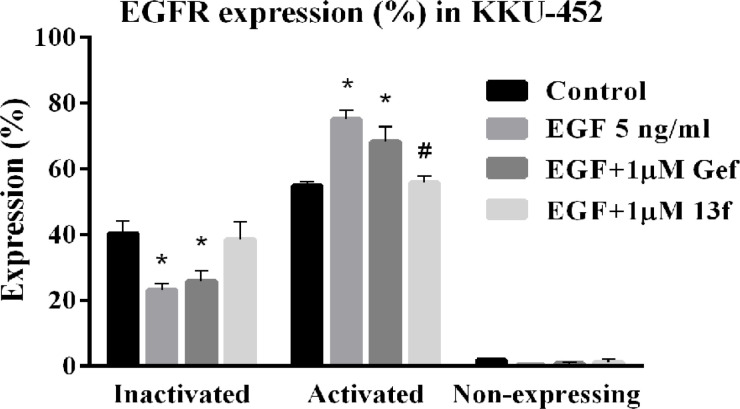
The effect of Gefitinib and 13f on Levels of EGFR Expression in CCA. KKU-452 cells were pretreated with 1 μM of Gefitinib and 1 μM of 13f, or a vehicle used as control for 3 h, and then with 5 ng/ ml of EGF for 24 h. Percentage of inactivated cells, activated cells (via EGFR phosphorylation) and non-expressing cells was shown for each condition. Data represent means ± SEM of three independent experiments. Value of *P < 0.05 vs control or # P < 0.05 vs EGF were indicated

Although 13f could inhibit cell proliferation, clonogenicity and migration of CCA cells, the effect of 13f on the EGFR expression remains unclear. An assessment of EGFR activation was performed using the Muse EGFR-RTK activation dual detection kit which detects phosphorylation of EGFR relative to total expression of EGFR in the cell population. KKU-452 cells were pretreated with 1 μM of Gefitinib and 1μM of 13f, or a vehicle treated control for 3 h, and then with 5 ng/ ml of EGF for 24 h. The level expression of EGFR was activated in KKU-452 cell with 55% under the basal level expression (Figure 5). After EGF-stimulated the level expression of activated EGFR was significantly increased with 75% when compared with control and significantly decreased after 13f treatment with 56% when compared with EGF alone. EGF-mediated activation was slightly decreased after Gefitinib treatment. The results suggest that EGFR pathway activation was found in CCA cell and 13f was almost against EGF-mediated activation. 

**Table 1:** Cytotoxicity of Gefininib, 13f and Cisplatin against KKU-100, KKU-452 and KKU-M156 Cells

Agents	KKU-100		KKU-452		KKU-M156	
	24 h	48 h	24 h	48 h	24 h	48 h
	IC50 (µM)	IC50 (µM)	IC50 (µM)	IC50 (µM)	IC50 (µM)	IC50 (µM)
Gefitinib	18.5 ± 11.6 *	4.0 ± 1.4	18.2 ± 6.8	14.9 ± 1.3	7.5 ± 3.2	4.5 ±0.6
13f	27.7 ± 9.7	1.3 ± 1.9	4.2 ± 2.2	1.5 ± 0.4	10.1 ± 6.3	1.7 ± 1.1
Cisplatin	38.4 ± 6.0	18.1 ± 3.9	63.6 ± 23.4	34.2 ± 14.1	33.5 ± 11.5	25.7 ± 5.2

* Each value is represented the mean ± SEM, each from three experiments (triplicate in each experiment)

## Discussion

EGFR pathway signaling is implicated for cancer genesis such as proliferation, chemotactic migration, invasion, and evasion of apoptosis (Burgess, 2008; Han and Lo, 2012). Activation of EGFR also triggers downstream MAPK–ERK intracellular signaling pathway. Mutations and amplifications in the *EGFR* gene have been found in 15% and 5% of CCA these abnormalities correlate with increased phosphorylation of downstream MAPK or Akt (Gwak et al., 2005; Nakazawa et al., 2005; Leone et al., 2006). Overexpression of EGFR has been associated with invasive CCA and poor prognosis (Yoshikawa et al., 2008; Harder et al., 2009). In this study our data found all CCA cell lines, KKU-100, KKU-452 and KKU-M156 cells, were resistant to Cisplatin but high response to Gefitinib and 13f. As EGFR is a promising target of CCA treatment we investigated the effect of 13f as a new synthetic of 4-aryl-N-phenylpyrimidin-2-amine on anti-cancer activities of CCA compared with Gefitinib as a traditional EGFR inhibitor.

Gefitinib is a small molecule of EGFR tyrosine kinase inhibitors (TKIs) used for second- or third-line treatment of advanced non-small cell lung cancer (de Marinis and Grossi, 2008; Tiseo et al., 2010). It binds to the adenosine triphosphate (ATP)-binding site of the intracellular tyrosine kinase and inhibits autophosphorylation consequent of blocking of EGFR signaling pathways (Giaccone, 2004). Gefitinib has been exerted anticancer activities including anti-proliferation, anti-apoptosis, and anti-angiogenesis in several human cancer cell lines with *EGFR* expression (Ciardiello et al., 2000; Ciardiello and Tortora, 2001; Sirotnak, 2003). Gefitinib has been shown to inhibit the proliferation of the CCC cell lines at high concentration (Nakajima et al., 2012). The effect of Gefitinib combination with Gemcitabine (pyrimidine analog) suppressed the proliferation of HuCCT1- and RBE- CCA cell lines by induction of apoptosis. This combination also has shown the synergistic effect of HuCCT1 xenografts in vivo (Nakajima et al., 2012). Thus, we used a Gefitinib for comparing the efficacy of anti-cancer effects with 13f a new EGFR inhibitor. In the present study, we demonstrated that 13f inhibited potential the tyrosine kinase of EGFR with the IC_50_ value is 22.74 nM. 13f is a more potent anti-cancer agent, which includes inhibition of proliferation by induced early apoptosis, inhibition of clonogenic ability and migration than Gefitinib by modulated EGFR signaling pathway in CCA. This data suggested that it is due to EGFR-TKI resistance. Previous report was found *T790M* mutation of the *EGFR *gene and MET amplification are known to be complicated in the major cases of acquired resistance to Gefitinib (Tiseo et al., 2010). But in lung cancer, the tyrosine kinase domain of the *EGFR* gene mutations was correlation with clinical efficacy of EGFR inhibitors (Leone et al., 2006). Moreover, activation of EGFR increases resistance to erlotinib EGFR-TKI inhibitor of CCA cells (Jimeno et al., 2005). In addition, HuCCT-1 (CCA cell line) cells now are resistant to Gefitinib and increase the sensitivity by combination treatment with CI-1040 which is an extracellular signal-regulated kinase (ERK) kinase 1/2 blocker. This combination treatment with Gefitinib and CI-1040 could be inhibited EGFR activation and block ERK1/2 phosphorylation downstream of EGFR pathway (Hidalgo et al., 2006). Our goal is to therefore to identify EGFR inhibitors with greater efficiency and specificity for CCA treatment 4-aryl-N-phenylpyrimidin-2-amine derivatives have been confirmed as EGFR inhibitors using a variety of methods discussed herein. Related compounds have also previously reported that target protein kinase (Pelletier et al., 2009; Crombie et al., 2010; Kamenecka et al., 2010), including at some downstream targets of EGFR. A new synthetic 13f or 3-[(4-phenylpyrimidin-2-yl)amino] benzene-1-sulfonamide is modification to the 2-position and the 4-position of the pyrimidine scaffold with 3-sulfonamido aniline at the R2, phenyl at the R1 (13f) (Toviwek et al., 2017). 13f show a good activity and induced the cytotoxicity in all CCA cells associated with previous reports. 13f has been evaluated the cytotoxicity in A549, NCI-H187, MCF7, Vero and KB cell lines) and found that potent anti-cancer activity of A549 with IC_50 _values of 0.2 µM compared to Doxorubicin drug (Toviwek et al., 2017). In this study we first discovered 13f also potent the cytotoxic effect to KKU-452 CCA cell with IC_50_ values of 4.2 µM at 24 h. and KKU-100 CCA cell with IC_50_ values of 1.3 µM at 48 h. The potential targets protein kinases of 13f may involve in the cytotoxicity were tested by computational methods that targeted including IKK2, GSK3, P38 JNK, CDK and EGFR (Toviwek et al., 2017). Moreover, we found 13f can induced cell death through early apoptosis pathway in a dose- and time dependence. We used Gefitinib as a positive control for apoptosis induction. The major apoptosis pathways include the extrinsic and intrinsic pathways. Apoptosis pathway is consistent with mechanism of action of anticancer agents; such as Gefitinib activated caspase-signaling apoptotic pathway, upregulated protein expression of BAX pro-apoptotic activity and induced cell cycle arrest (Yan et al., 2015), Cisplatin can suppress the Bcl-xl antiapoptosis protein (Samatiwat et al., 2016). However, the mechanism of 13f induced apoptosis are not evaluated. We need to further exploration the imbalance of proapoptotic and antiapoptosis probably related to 13f enhance CCA cell sensitivity. 

In vitro study needs to clarify the role of their targets and inhibition levels for anticancer targeted therapy. We found the constitutive expression and activation of EGFR signaling pathway which could be enhance the activated phosphorylation by EGF in CCA. Another study also revealed EGFR signaling activation and indicated that expressions of EGFR mRNA and EGFR phosphorylation were high level in HuCCT1 and RBE human CCC cell lines (Nakajima et al., 2012). Previous report found that 15% CCA have tyrosine kinase domain of *EGFR* gene mutations and activate EGFR downstream pathways when compared with EGFR wildtype cancer (Leone et al., 2006). Activation of EGFR signaling pathway may involve with *EGFR* mutation in CCA. Base on molecular docking of 13f can bind to the EGFR pocket, we determined the precise inhibition of EGFR targeted for 13f of CCA. 13f at low concentration could suppress EGF-mediated activation of EGFR phosphorylation and consequence of proliferation-, clonogenic ability- and migration activity- inhibition in CCA. Previous study of combination treatment of Gefitinib and gemcitabine enhance the cytotoxicity could reduce the protein of ERK phosphorylation and indicated for EGFR signaling suppression in CCA cell (Nakajima et al., 2012). Downstream of EGFR signaling pathways include the MAP kinase cascade that activates several genes imply to cell survival and proliferation, and PI3K-AKT cascade that phosphorylated AKT can inactivate proapoptotic proteins (Cappuzzo et al., 2004; Wee and Wang, 2017). 

In conclusion, our findings suggest that the 4-aryl-N-phenylpyrimidin-2-amine 13f acts at EGFR kinase and resulting in potent anti-cancer activity in CCA cells. The strategy of targeting EGFR proved highly successful for treating a range of cancers and the identification of new scaffold with interesting activity is important in the development of new therapies to overcome disease resistance (Patel et al., 2017).
